# Supplementary material for: Resolving the full spectrum of human genome variation using Linked-Reads
Source: Genome Res. 2019 Apr;29(4):635–45. doi: 10.1101/gr.234443.118 (PMC6442396; doi:10.1101/gr.234443.118)
Supplement: Supplemental Material [file supp_gr.234443.118_Supplemental_longranger-2.2.zip › longranger-2.2.tar.bz2!/lr2.2-public/lib/stan/cmdstan/stan/doc/api/html/bare__type__grammar_8hpp_source.html]

Stan: src/stan/gm/grammars/bare\_type\_grammar.hpp Source File


|  |  |
| --- | --- |
|  | Stan  2.5.0  probability, sampling & optimization |


- Main Page
- Related Pages
- Namespaces
- Classes
- Files

- File List
- File Members

All Classes Namespaces Files Functions Variables Typedefs Enumerations Enumerator Friends Macros Pages

- src
- stan
- gm
- grammars

bare\_type\_grammar.hpp

Go to the documentation of this file.

1 #ifndef STAN\_\_GM\_\_PARSER\_\_BARE\_TYPE\_GRAMMAR\_HPP\_\_

2 #define STAN\_\_GM\_\_PARSER\_\_BARE\_TYPE\_GRAMMAR\_HPP\_\_

3

4 #include <string>

5 #include <sstream>

6 #include <vector>

7

8 #include <boost/spirit/include/qi.hpp>

9

10 #include <stan/gm/ast.hpp>

11 #include <stan/gm/grammars/whitespace\_grammar.hpp>

12 #include <stan/gm/grammars/statement\_grammar.hpp>

13

14 namespace stan {

15

16  namespace gm {

17

18  template <typename Iterator>

19  struct bare\_type\_grammar

20  : boost::spirit::qi::grammar<Iterator,

21  expr\_type(),

22  whitespace\_grammar<Iterator> > {

23

24  // global info for function defs

25  variable\_map& var\_map\_;

26  std::stringstream& error\_msgs\_;

27

28  // constructor

29  bare\_type\_grammar(variable\_map& var\_map,

30  std::stringstream& error\_msgs);

31

32  boost::spirit::qi::rule<Iterator,

33  expr\_type(),

34  whitespace\_grammar<Iterator> >

35  bare\_type\_r;

36

37  boost::spirit::qi::rule<Iterator,

38  base\_expr\_type(),

39  whitespace\_grammar<Iterator> >

40  type\_identifier\_r;

41

42  boost::spirit::qi::rule<Iterator,

43  size\_t(),

44  whitespace\_grammar<Iterator> >

45  array\_dims\_r;

46

47  };

48  }

49 }

50

51 #endif

stan::gm::bare\_type\_grammar::bare\_type\_grammar

bare\_type\_grammar(variable\_map &var\_map, std::stringstream &error\_msgs)

**Definition:** bare\_type\_grammar\_def.hpp:37

stan::gm::bare\_type\_grammar::array\_dims\_r

boost::spirit::qi::rule< Iterator, size\_t(), whitespace\_grammar< Iterator > > array\_dims\_r

**Definition:** bare\_type\_grammar.hpp:45

stan::gm::bare\_type\_grammar::bare\_type\_r

boost::spirit::qi::rule< Iterator, expr\_type(), whitespace\_grammar< Iterator > > bare\_type\_r

**Definition:** bare\_type\_grammar.hpp:35

statement\_grammar.hpp

stan::gm::bare\_type\_grammar::type\_identifier\_r

boost::spirit::qi::rule< Iterator, base\_expr\_type(), whitespace\_grammar< Iterator > > type\_identifier\_r

**Definition:** bare\_type\_grammar.hpp:40

stan::gm::variable\_map

**Definition:** ast.hpp:439

stan::gm::expr\_type

**Definition:** ast.hpp:74

stan::gm::bare\_type\_grammar::var\_map\_

variable\_map & var\_map\_

**Definition:** bare\_type\_grammar.hpp:25

ast.hpp

stan::gm::base\_expr\_type

int base\_expr\_type

**Definition:** ast.hpp:60

stan::gm::whitespace\_grammar

**Definition:** whitespace\_grammar.hpp:11

whitespace\_grammar.hpp

stan::gm::bare\_type\_grammar

**Definition:** bare\_type\_grammar.hpp:19

stan::gm::bare\_type\_grammar::error\_msgs\_

std::stringstream & error\_msgs\_

**Definition:** bare\_type\_grammar.hpp:26


---

[ Stan Home Page ]

*© 2011–2014,
Stan Development Team.*
